# Antibiotics in Orthopedic Infections

**DOI:** 10.3390/antibiotics10111297

**Published:** 2021-10-25

**Authors:** Konstantinos Anagnostakos, Bernd Fink

**Affiliations:** 1 Zentrum für Orthopädie und Unfallchirurgie, Klinikum Saarbrücken, 66119 Saarbrücken, Germany; 2 Department for Joint Replacement, Rheumatoid and General Orthopaedics, Orthopaedic Clinic Markgröningen, Kurt-Lindemann Weg 10, 71706 Markgröningen, Germany; 3 Orthopaedic Department, University Hospital Hamburg-Eppendorf, Martinistrasse 52, 20251 Hamburg, Germany

The management of orthopedic infections has continuously been gaining increasing interest in the past few years. Various developments of new techniques have enhanced pre- and intraoperative diagnostics, leading to an increased number of identified pathogen organisms in septic but also assumed aseptic revisions. In addition to surgical debridement, knowledge about the systemic and local antibiotic therapy of bone and joint infections is an indispensable premise for a successful outcome. This Special Issue deals with all these topics and more, and includes research articles as well as a review. 

The enhancement of microbiological detection techniques has led to an increasing number of organisms at the site of periprosthetic joint infections (PJI). Anagnostakos et al. evaluated the frequency and antibiotic resistance profiles of rare pathogens at the site of hip and knee PJIs [1]. These organisms accounted for almost 10% of all infections. Most of these pathogens were multi-susceptible to the tested antibiotics. The authors concluded that no specific adjustment of the systemic antibiotic therapy is required in these cases. Darwich et al. assessed the outcome of PJIs with superinfections with a difficult-to-treat (DTT) pathogen [2]. PJI caused initially by a non-DTT pathogen with a superinfection with a DTT pathogen was significantly associated with the worst outcome in comparison to non-DTT-PJI, PJI caused initially by a DTT pathogen, and to non-DTT-PJI with a non-DTT superinfection.

Despite the improvement made to microbiological techniques, there still exists no gold standard method with a 100% sensitivity and specificity. Based on this problem, Fink et al. described a graphical representation of cell count as a new technique in PJI diagnosis [3]. At the site of 322 cases, a significant correlation between the graphical matrices of synovial cell counting and the histological types of Morawietz and Krenn was found. This new approach might help to increase the diagnostic value of cell count analysis in the diagnosis of PJI and specifically distinguish between elevated cell counts in the automatic analysis because of infection and debris wear. 

Regarding the systemic therapy of PJI, rifampicin is accepted as being a valuable adjunct due to its biofilm activity. Rifampicin resistance might therefore have a devastating effect on the clinical outcome. Darwich et al. evaluated the incidence of rifampicin resistance between two groups of patients with a PJI treated with antibiotic regimens involving either immediate or delayed additional rifampicin administration and the effect of this resistance on the outcome [4]. A significant association between the immediate start of rifampicin after surgical revision in the treatment of PJI and the emergence of rifampicin resistance was found, but with no significant effect on outcome.

In addition to systemic antibiotic therapy, local antibiotic therapy by means of spacers or beads is also crucial in the successful management of PJIs. Regarding the production of custom-made hip spacers, Mederake et al. presented a new technique [5]. In a cohort of 130 patients, the infection eradication rate was 92% at a median follow-up of 51 months. Spacer-related complications were observed in 10% of the cases. In another study by Anagnostakos et al., the antibiotic impregnation of hip and knee spacers and beads was investigated in relation to the resistance profiles of the causative organisms and the infectiological outcome [6]. Complete susceptibility was present in 38.7% of the cases and partial susceptibility in 28%. In the remaining 33.3%, no precise statement could be made, because either there was a culture-negative infection or the antibiotics were not tested against the specific organism. Treatment failure was observed in 6.7% of the cases. Independent of which antibiotic impregnation was used, when the organism was susceptible against the locally inserted antibiotics or not tested, reinfection or persistence of infection was observed in the great majority of cases. Since these findings cannot be solely explained by the interpretation of the antibiotic resistance profiles, the optimal impregnation of antibiotic-loaded bone cement should be further investigated in future studies. 

Tuecking et al. performed a detailed revision risk analysis after single- vs. two-stage revision surgery in the management of knee PJIs [7]. No significant difference was found between both groups in overall and implant survival with respect to reinfection. High reinfection rates were found for patients with DTT organisms and low- to semi-constrained implant types, in comparison to constrained implant types. A statistically comparable revision rate for recurrence of infection could be shown for both groups, although a tendency toward a higher reinfection rate for single-stage change was evident. Bdeir et al. described their experience with the management of shoulder PJIs by use of an algorithm initially established for hip and knee PJIs [8]. The treatment failure rate was 10.5%. After interpretation of these findings, the authors suggested that therapeutical algorithms and recommendations developed for the treatment of PJI of the hip and knee are also applicable to the shoulder joint.

Local antibiotic therapy is not only of advantage at the site of PJIs, but also in the management of septic arthritis of native joints. In an in vitro model, Papalia et al. reported on the possible effect of vancomycin on the activity of tenocytes at the site of anterior cruciate ligament reconstruction [9]. After testing different concentrations of vancomycin over various time periods with regard to metabolic activity, cell toxicity and apoptosis, vancomycin was found to be useful and safe, if used at a concentration of 2.5 mg/mL for up to one hour of treatment.

In their review work, Goh and Parvizi reported on the influence of antibiotics on the diagnosis of PJI [10]. The effect of prophylactic and therapeutic antibiotic administration on the diagnostic accuracy of microbiological cultures and serum or synovial biomarkers is presented. Of interest is the fact that preoperative antibiotic administration seems to have a negligible effect on the sensitivity of more recent biomarkers such as alpha defensin and leukocyte esterase. Newer molecular techniques, such as 16S rRNA gene sequencing or metagenomic next generation sequencing (mNGS), might pose a solution in enhancing the sensitivity of microbiological diagnostics in the future; however, these methods have not yet gained widespread adoption.

Within this Special Issue, all authors have done a great job and provided high-quality articles. We hope that this Special Issue will not only provide some new information about orthopedic infections, but also encourage our colleagues to carry on with clinical and scientific work in order to further enhance the treatment of our patients.

## References

[1] Anagnostakos K., Grzega C., Sahan I., Geipel U., Becker S.L. (2021). Occurrence of rare pathogens at the site of periprosthetic hip and knee joint infections: A retrospective, single-center study. Antibiotics.

[2] Darwich A., Dally F.J., Abu Olba K., Mohs E., Gravius S., Hetjens S., Assaf A., Bdeir M. (2021). Superinfection with difficult-to-treat pathogens significantly reduces the outcome of periprosthetic joint infections. Antibiotics.

[3] Fink B., Hoyka M., Weissbarth E., Schuster P., Berger I. (2021). The graphical representation of cell count representation: A new procedure for the diagnosis of periprosthetic joint infections. Antibiotics.

[4] Darwich A., Dally F.J., Bdeir M., Kehr K., Miethke T., Hetjens S., Gravius S., Assaf E., Mohs E. (2021). Delayed rifampin administration in the antibiotic treatment of periprosthetic joint infections significantly reduces the emergence of rifampin resistance. Antibiotics.

[5] Mederake M., Hofmann U.K., Fink B. (2021). New technique for custom-made spacers in septic two-stage revision of total hip arthroplasties. Antibiotics.

[6] Anagnostakos K., Sahan I. (2021). Are cement spacers and beads loaded with the correct antibiotic(s) at the site of periprosthetic hip and knee joint infections?. Antibiotics.

[7] Tuecking L.R., Silligmann J., Savov P., Omar M., Windhagen W., Ettinger M. (2021). Detailed revision risk analysis after single- vs. two-stage revision total knee arthroplasty in periprosthetic joint infection: A retrospective tertiary center analysis. Antibiotics.

[8] Bdeir M., Dally F.J., Assaf E., Gravius S., Mohs E., Hetjens S., Darwich A. (2021). Periprosthetic infections of the shoulder joint: Characteristics and 5-year outcome of a single-center series of 19 cases. Antibiotics.

[9] Papalia R., Cicione C., Russo F., Ambrosio L., Di Giacomo G., Vadala G., Denaro V. (2021). Does vancomycin wrapping in anterior cruciate ligament reconstruction affect tenocyte activity in vitro?. Antibiotics.

[10] Goh G.S., Parvizi J. (2021). Think twice before prescribing antibiotics for that swollen knee: The influence of antibiotics on the diagnosis of periprosthetic joint infection. Antibiotics.

